# Supplementary material for: Clinical outcomes and patient-reported measures in HCV care: Insight from a longitudinal prospective study in a large Italian region
Source: PLoS One. 2026 Mar 11;21(3):e0343936. doi: 10.1371/journal.pone.0343936 (PMC12978477; doi:10.1371/journal.pone.0343936)
Supplement: S1 File — Sociodemographic characteristics, clinical history, referral pathways, patient-reported experience measures (PREMs), and patient-reported outcome measures (PROMs) of HCV patients, stratified by referral source (general practitioners, Harm Reduction/prison services, opportunistic/specialist doctors) and time point. (DOCX) [file pone.0343936.s001.docx]

**SUPPLEMENTARY MATERIALS – TABLES**

|  |  | Whole Population | A.  General Practitioner | A  vs  B | B. SERD/Prison Doctor | B  vs  C | C. Opportunistic/ Specialist Doctor | C  vs  A | Overall Analysis  (one way ANOVA/Kruskal-Wallis analysis) |
| --- | --- | --- | --- | --- | --- | --- | --- | --- | --- |
|  | n | 347 | 216 |  | 55 |  | 66 |  |  |
| **Socio-demographic characteristics** | | | | | | | | | |
| *Age (years,* Mean±SD*)* | | 58.21±15.95 | 60.51±15.70 | **<0.0005** | 45.89±11.19 | **<0.0005** | 60.41±16.23 | 1.000 | **F(2.334)=21.297**  **p<0.0005** |
| *Gender* | Males (%) | 59.9 | 55.6 | **<0.0005** | 90.9 | **<0.0005** | 50.0 | 1.000 | χ2(2)=26.388 p<0.0005 |
|  | Female (%) | 40.1 | 44.4 |  | 9.1 |  | 50.0 |  |  |
|  | n | 347 | 216 |  | 55 |  | 66 |  |  |
| *Work Occupation* | Student (%) | 1.6 | 1.0 |  | 0.0 |  | 4.9 |  | χ2(2)=5.280 p=0.071 |
|  | Employee (%) | 35.6 | 36.6 |  | 39.2 |  | 32.8 |  | χ2(2)=0.521 p=0.771 |
|  | Freelancer (%) | 7.7 | 7.9 |  | 3.9 |  | 9.8 |  | χ2(2)=1.431 p=0.489 |
|  | Entrepreneur (%) | 2.9 | 3.1 |  | 0.0 |  | 4.9 |  | χ2(2)=2.376 p=0.305 |
|  | Retired (%) | 34.0 | 40.8 | **<0.0005** | 9.8 | 0.052 | 31.1 | 0.492 | **χ2(2)=17.518 p<0.0005** |
|  | Unemployed (%) | 18.3 | 10.5 | **<0.0005** | 47.1 | **<0.0005** | 16.4 | 0.880 | **χ2(2)=36.776 p<0.0005** |
|  | Overall Analysis |  |  | **0.049** |  | 0.138 |  | 1.000 | **χ2(2)=6.079 p=0.048** |
|  | n | 312 | 191 |  | 51 |  | 61 |  |  |
| *Education Level* | No Title/Elementary School Diploma (%) | 15.8 | 18.4 |  | 5.9 |  | 12.3 |  |  |
|  | Middle School Diploma (%) | 44.8 | 42.2 |  | 62.7 |  | 43.1 |  |  |
|  | High School Diploma (%) | 28.2 | 27.7 |  | 27.5 |  | 29.2 |  |  |
|  | Degree (%) | 9.7 | 9.2 |  | 3.9 |  | 15.4 |  |  |
|  | Post-graduate Qualification (%) | 1.5 | 2.4 |  | 0.0 |  | 0.0 |  |  |
|  | Overall Analysis |  |  |  |  |  |  |  | χ2(2)=1.469 p=0.480 |
|  | n |  | 206 |  | 51 |  | 65 |  |  |
| *Birth Area* | Italy (%) | 88.9 | 88.3 |  | 90.7 |  | 90.9 |  |  |
|  | Europe (%) | 7.9 | 8.5 |  | 7.4 |  | 4.5 |  |  |
|  | Asia (%) | 1.5 | 0.5 |  | 0.0 |  | 0.0 |  |  |
|  | Africa (%) | 1.2 | 0.0 |  | 0.0 |  | 1.5 |  |  |
|  | Nord America (%) | 0.3 | 1.9 |  | 0.0 |  | 1.5 |  |  |
|  | Sud America (%) | 0.3 | 0.9 |  | 1.9 |  | 1.5 |  |  |
|  | Overall Analysis |  |  |  |  |  |  |  | χ2(2)=0.481  p=0.786 |
|  | n | 342 | 213 |  | 54 |  | 66 |  |  |
| Italian Regions | Nord Italy (%) | 9.8 | 9.8 |  | 10.4 |  | 10.3 |  |  |
|  | Central Italy (%) | 72.9 | 72.8 |  | 70.8 |  | 74.1 |  |  |
|  | Sud Italy (%) | 17.3 | 17.4 |  | 18.8 |  | 15.5 |  |  |
|  | Overall Analysis |  |  |  |  |  |  |  | χ2(2)=0.129 p=0.937 |
|  | n | 295 | 184 |  | 48 |  | 58 |  |  |
| **HCV Risk Factors** | | | | | | | | | |
| *Blood Transfusions, Haemodialysis, Transplants before 90s* | Yes (%) | 18.3 | 20.2 | **0.040** | 5.6 | **0.038** | 23.4 | 1.000 | **χ2(2)=7.437**  **p=0.024** |
|  | No (%) | 81.7 | 79.8 |  | 94.4 |  | 76.6 |  |  |
|  | n | 338 | 213 |  | 54 |  | 64 |  |  |
| *Blood Donor before 90s* | Yes (%) | 8.8 | 10.0 |  | 3.6 |  | 9.1 |  | χ2(2)=2.190  p=0.335 |
|  | No (%) | 91.2 | 90.0 |  | 96.4 |  | 90.9 |  |  |
|  | n | 340 | 211 |  | 55 |  | 66 |  |  |
| *Injection Drug Use* | Yes (%) | 34.5 | 24.1 | **0.005** | 90.6 | **0.005** | 24.2 | 1.000 | **χ2(2)=86.163**  **p<0.0005** |
|  | No (%) | 65.5 | 75.9 |  | 9.4 |  | 75.8 |  |  |
|  | n | 336 | 212 |  | 53 |  | 62 |  |  |
| *Tattoo/Piercing in Environments with Uncertain Sterility* | Yes (%) | 23.7 | 21.4 | 0.051 | 37.5 | **0.022** | 15.9 | 1.000 | **χ2(2)=7.821**  **p=0.020** |
|  | No (%) | 76.3 | 78.6 |  | 62.5 |  | 84.1 |  |  |
|  | n | 321 | 201 |  | 48 |  | 63 |  |  |
| Sharing Toothbrush or Razor Blades | Yes (%) | 17.6 | 17.3 |  | 26.9 |  | 11.3 |  | χ2(2)=4.799  p=0.091 |
|  | No (%) | 82.4 | 82.7 |  | 73.1 |  | 88.7 |  |  |
|  | n | 319 | 197 |  | 52 |  | 62 |  |  |
| Unprotected Sexual Contact | Yes (%) | 68.7 | 63.0 | **<0.0005** | 96.1 | **0.001** | 64.5 | 1.000 | **χ2(2)=21.315**  **p<0.0005** |
|  | No (%) | 31.3 | 37.0 |  | 3.9 |  | 35.5 |  |  |
|  | n | 329 | 208 |  | 51 |  | 62 |  |  |
| Mother with HCV when HCV Patient was Born | Yes (%) | 2.8 | 2.3 |  | 2.0 |  | 6.0 |  | χ2(2)=2.131  p=0.344 |
|  | No (%) | 97.2 | 97.7 |  | 98.0 |  | 94.0 |  |  |
|  | n | 287 | 177 |  | 51 |  | 50 |  |  |
| Previously Detention Periods | Yes (%) | 17.5 | 11.7 | **<0.0005** | 47.3 | **<0.0005** | 13.8 | 1.000 | **χ2(2)=38.205**  **p<0.0005** |
|  | No (%) | 82.5 | 88.3 |  | 52.7 |  | 86.2 |  |  |
|  | n | 343 | 213 |  | 55 |  | 65 |  |  |
| Actual Detention Periods | Yes (%) | 2.6 | 2.4 | 0.138 | 7.3 | **0.044** | 0.0 | 0.919 | **χ2(2)=6.232**  **p=0.044** |
|  | No (%) | 97.4 | 97.6 |  | 92.7 |  | 100.0 |  |  |
|  | n | 342 | 212 |  | 55 |  | 65 |  |  |
| Other Pathologies | Yes (%) | 48.5 | 51.2 | **0.007** | 27.8 | **0.003** | 58.7 | 0.881 | **χ2(2)=12.480**  **p=0.002** |
|  | No (%) | 51.5 | 48.8 |  | 72.2 |  | 41.3 |  |  |
|  | n | 338 | 211 |  | 54 |  | 63 |  |  |
| Diabete | Yes (%) | 22.2 | 21.3 |  | 20.0 |  | 24.3 |  | χ2(2)=0.181  p=0.914 |
|  | No (%) | 78.0 | 78.7 |  | 80.0 |  | 75.7 |  |  |
|  | n | 164 | 108 |  | 15 |  | 37 |  |  |
| HBV | Yes (%) | 3.7 | 3.7 |  | 0.0 |  | 5.4 |  | χ2(2)=0.861  p=0.650 |
|  | No (%) | 96.3 | 96.3 |  | 100.0 |  | 94.6 |  |  |
|  | n | 164 | 108 |  | 15 |  | 37 |  |  |
| HIV | Yes (%) | 3.0 | 1.9 |  | 6.7 |  | 5.4 |  | χ2(2)=1.824  p=0.402 |
|  | No (%) | 97.0 | 98.1 |  | 93.3 |  | 94.6 |  |  |
|  | n | 164 | 108 |  | 15 |  | 37 |  |  |
| Cardiovascular Diseases | Yes (%) | 36.0 | 42.6 | 0.083 | 13.3 | 1.000 | 27.0 | 0.271 | **χ2(2)=6.609**  **p=0.037** |
|  | No (%) | 64.0 | 57.4 |  | 86.7 |  | 73.0 |  |  |
|  | n | 164 | 108 |  | 15 |  | 37 |  |  |
| Endocrine Diseases | Yes (%) | 7.9 | 9.3 |  | 0.0 |  | 8.1 |  | χ2(2)=1.503  p=0.472 |
|  | No (%) | 92.1 | 90.7 |  | 100.0 |  | 91.9 |  |  |
|  | n | 164 | 108 |  | 15 |  | 37 |  |  |
| Lung Diseases | Yes (%) | 8.5 | 6.5 |  | 20.0 |  | 10.8 |  | χ2(2)=3.250  p=0.197 |
|  | No (%) | 91.5 | 93.5 |  | 80.0 |  | 89.2 |  |  |
|  | n | 164 | 108 |  | 15 |  | 37 |  |  |
| Neurologic Diseases | Yes (%) | 9.8 | 10.2 |  | 6.7 |  | 8.1 |  | χ2(2)=0.281  p=0.869 |
|  | No (%) | 90.2 | 89.8 |  | 93.3 |  | 91.9 |  |  |
|  | n | 164 | 108 |  | 15 |  | 37 |  |  |
| Psychiatric Diseases | Yes (%) | 5.5 | 2.8 | **<0.0005** | 33.3 | **<0.0005** | 2.7 | 1.000 | **χ2(2)=23.788**  **p<0.0005** |
|  | No (%) | 94.5 | 97.2 |  | 66.7 |  | 97.3 |  |  |
|  | n | 164 | 108 |  | 15 |  | 37 |  |  |
| Autoimmune Diseases | Yes (%) | 9.8 | 7.4 | 1.000 | 0.0 | 0.057 | 21.6 | **0.039** | **χ2(2)=7.976**  **p=0.019** |
|  | No (%) | 90.2 | 92.6 |  | 100.0 |  | 78.4 |  |  |
|  | n | 164 | 108 |  | 15 |  | 37 |  |  |
| Oncologic Diseases | Yes (%) | 13.4 | 13.0 |  | 6.7 |  | 16.2 |  | χ2(2)=0.856  p=0.652 |
|  | No (%) | 86.6 | 87.0 |  | 93.3 |  | 83.8 |  |  |
|  | n | 164 | 108 |  | 15 |  | 37 |  |  |

**Supplementary Table 1.** *Sociodemographic characteristics and clinical history of the HCV patients that filled the questionnaire at T0.* Data refer to whole population, as well as to relative groups arriving to HCV diagnosis through general (A), SERD/prison (B) or specialist (C) physicians. Comparisons between different groups were performed by Kruskal-Wallis analysis. When the overall analysis was statistically significant, a Kruskal-Wallis test was applied again for each category represented.

|  |  | Whole Population | A.  General Practitioner | A  vs  B | B. SERD/Prison Doctor | B  vs  C | C. Opportunistic/ Specialist Doctor | C  vs  A | Overall Analysis  (one way ANOVA/ Kruskal-Wallis analysis) |
| --- | --- | --- | --- | --- | --- | --- | --- | --- | --- |
|  | n | 347 | 216 |  | 55 |  | 66 |  |  |
| ***Referring Doctor - Diagnosis and Behavior*** | | | | | | | | | |
|  |  |  |  |  |  |  |  |  |  |
|  |  |  |  |  |  |  |  |  |  |
| *HCV Discovery: When* | < 1 month (%) | 6.3 | 4.8 | 1.000 | 1.9 | **0.046** | 12.3 | 0.073 | **χ2(2)=6.921**  **p=0.031** |
|  | 1-3 months (%) | 17.4 | 16.3 | 0.666 | 9.3 | **0.025** | 27.7 | 0.107 | **χ2(2)=7.408**  **p=0.025** |
|  | 3-6 months (%) | 6.6 | 6.3 |  | 1.9 |  | 12.3 |  | χ2(2)=5.330  p=0.070 |
|  | 6-12 months (%) | 8.1 | 7.2 |  | 9.3 |  | 9.2 |  | χ2(2)=0.426  p=0.808 |
|  | > 12 months (%) | 61.7 | 65.4 | 0.285 | 77.8 | **<0.0005** | 38.5 | **<0.0005** | **χ2(2)=21.953**  **p<0.0005** |
|  | Overall Analysis |  |  | 0.209 |  | **<0.0005** |  | **<0.0005** | **χ2(2)=24.001**  **p<0.0005** |
|  | n | 334 | 208 |  | 54 |  | 65 |  |  |
| *HCV Discovery: How* | Routine Blood Tests (%) | 84.6 | 89.0 | **<0.0005** | 56.3 | **<0.0005** | 97.4 | 0.561 | **χ2(2)=36.494**  **p<0.0005** |
|  | Checks requested to the referring doctor: tattoos/piercing were carried out in unsafe environments (%) | 4.4 | 3.3 |  | 10.4 |  | 2.6 |  | χ2(2)=4.846  p=0.089 |
|  | Routine Blood Tests Carried Out due to Drug Addiction (%) | 7.0 | 2.2 | **<0.0005** | 31.3 | **<0.0005** | 0.0 | 1.000 | **χ2(2)=51.862**  **p<0.0005** |
|  | “TestiamoCi” Screening (%) | 0.7 | 1.1 |  | 0.0 |  | 0.0 |  | χ2(2)=0.965  p=0.617 |
|  | Medical Exams for Preventive Medicine during Work (%) | 3.3 | 4.4 |  | 2.1 |  | 0.0 |  | χ2(2)=2.216  p=0.330 |
|  | Overall Analysis |  |  | **<0.0005** |  | **<0.0005** |  | 0.484 | **χ2(2)=34.503**  **p<0.0005** |
|  | n | 273 | 181 |  | 48 |  | 39 |  |  |
| *Ease of Access to Primary Care* | Yes (%) | 91.9 | 91.5 |  | 92.6 |  | 91.8 |  | χ2(2)=0.062  p=0.969 |
|  | No (%) | 8.1 | 8.5 |  | 7.4 |  | 8.2 |  |  |
|  | n | 322 | 201 |  | 54 |  | 61 |  |  |
| *After First Suspicion, In-Depth Tests for HCV are Prescribed by the Referring Doctor* | Yes (%) | 80.2 | 85.3 | 1.000 | 83.3 | **0.013** | 62.3 | **<0.0005** | **χ2(2)=16.317**  **p<0.0005** |
|  | No (%) | 19.8 | 14.7 |  | 16.7 |  | 37.7 |  |  |
|  | n | 334 | 211 |  | 54 |  | 61 |  |  |
| *Time between HCV Specific Analysis and Results* | > 1 week (%) | 57.5 | 54.0 |  | 70.4 |  | 56.3 |  |  |
|  | 5-7 days (%) | 19.7 | 21.7 |  | 18.5 |  | 17.2 |  |  |
|  | 3-5 days (%) | 14.5 | 18.2 |  | 5.6 |  | 10.9 |  |  |
|  | 1-2 days (%) | 8.3 | 6.1 |  | 5.6 |  | 15.6 |  |  |
|  | Overall Analysis |  |  |  |  |  |  |  | χ2(2)=5.240  p=0.073 |
|  | n | 325 | 198 |  | 54 |  | 64 |  |  |
| *HCV Diagnosis, What the Referring Doctor Did* | Patient was referred to a specialist facility. The doctor has booked an appointment directly (%) | 61.7 | 55.6 | 0.371 | 68.2 | 0.758 | 80.0 | **0.008** | **χ2(2)=10.041**  **p=0.007** |
|  | Patient was referred to a specialist facility. Appointment was booked directly by the patient (%) | 29.8 | 34.2 | 1.000 | 27.3 | 0.687 | 15.6 | **0.043** | **χ2(2)=6.184**  **p=0.045** |
|  | The doctor recommended the HCV patients to look for a specialist centre and to make an appointment (%) | 8.5 | 10.2 |  | 4.5 |  | 4.4 |  | χ2(2)=2.525  p=0.283 |
|  | Overall Analysis |  |  | 0.302 |  | 0.907 |  | **0.009** | **χ2(2)=10.036**  **p=0.007** |
|  | n | 282 | 187 |  | 44 |  | 45 |  |  |
| ***Care Pathway in Specialist Medicine*** | | | | | | | | | |
|  |  |  |  |  |  |  |  |  |  |
|  |  |  |  |  |  |  |  |  |  |
| *Treatment Path: Type of Specialist Doctor* | Infectious disease specialist (%) | 38.9 | 35.9 |  | 52.2 |  | 40.3 |  |  |
|  | Hepatologist (%) | 56.7 | 60.1 |  | 41.3 |  | 54.8 |  |  |
|  | Gastroenterologist (%) | 2.2 | 1.5 |  | 2.2 |  | 4.8 |  |  |
|  | Other Specialist (%) | 2.2 | 2.5 |  | 4.3 |  | 0.0 |  |  |
|  | Overall Analysis |  |  |  |  |  |  |  | χ2(2)=2.916  p=0.233 |
|  | n | 314 | 198 |  | 46 |  | 62 |  |  |
| First Specialist Doctor Visit: Time Elapsed from the Call | > 1 months (%) | 12.3 | 12.6 |  | 11.5 |  | 13.3 |  |  |
|  | 1 months (%) | 21.2 | 20.9 |  | 32.7 |  | 15.0 |  |  |
|  | 2 weeks (%) | 28.2 | 31.1 |  | 28.8 |  | 20.0 |  |  |
|  | 1 week (%) | 38.3 | 35.4 |  | 26.9 |  | 51.7 |  |  |
|  | Overall Analysis |  |  |  |  |  |  |  | χ2(2)=5.199  p=0.074 |
|  | n | 326 | 206 |  | 52 |  | 60 |  |  |
| Appointment in the Most Convenient Specialist Location | Yes (%) | 94.9 | 95.7 | 0.106 | 88.7 | **0.048** | 98.4 | 1.000 | **χ2(2)=6.283**  **p=0.043** |
|  | No (%) | 5.1 | 4.3 |  | 11.3 |  | 1.6 |  |  |
|  | n | 334 | 209 |  | 53 |  | 63 |  |  |
| Easy access to specialist care | 1_Strongly Disagree (%) | 2.1 | 2.4 |  | 3.8 |  | 0.0 |  |  |
|  | 2_Disagree (%) | 3.3 | 3.4 |  | 3.8 |  | 3.2 |  |  |
|  | 3_Undecided (%) | 6.3 | 5.8 |  | 9.4 |  | 6.5 |  |  |
|  | 4_Agree (%) | 13.9 | 14.9 |  | 20.8 |  | 6.5 |  |  |
|  | 5_Strongly Agree (%) | 74.3 | 73.6 |  | 62.3 |  | 83.9 |  |  |
|  | Mean Ranks |  | 161.95 | 0.299 | 143.68 | **0.034** | 177.84 | 0.384 | **χ2(2)=6.407**  **p=0.041** |
|  | Mean±SD | 4.55±0.91 | 4.54±0.92 |  | 4.34±1.06 |  | 4.71±0.73 |  |  |
|  | n | 331 | 208 |  | 53 |  | 62 |  |  |
| Specialist Doctor during the First Visit:  Vision of the Medical Exams Brought by the Patients | Yes (%) | 77.2 | 80.1 |  | 69.1 |  | 74.2 |  | χ2(2)=3.394  p=0.183 |
|  | No (%) | 22.8 | 19.9 |  | 30.9 |  | 25.8 |  |  |
|  | n | 347 | 216 |  | 55 |  | 66 |  |  |
| Specialist Doctor during the First Visit:  Visit of the Patient | Yes (%) | 51.9 | 56.5 |  | 45.5 |  | 42.4 |  | χ2(2)=5.090  p=0.078 |
|  | No (%) | 48.1 | 43.5 |  | 54.5 |  | 57.6 |  |  |
|  | n | 347 | 216 |  | 55 |  | 66 |  |  |
| Specialist Doctor during the First Visit:  Echography | Yes (%) | 37.2 | 34.7 |  | 41.8 |  | 36.4 |  | χ2(2)=0.954  p=0.621 |
|  | No (%) | 62.8 | 65.3 |  | 58.2 |  | 63.6 |  |  |
|  | n | 347 | 216 |  | 55 |  | 66 |  |  |
| Specialist Doctor during the First Visit:  Elastography | Yes (%) | 17.9 | 18.1 |  | 12.7 |  | 19.7 |  | χ2(2)=1.131  p=0.568 |
|  | No (%) | 82.1 | 81.9 |  | 87.3 |  | 80.3 |  |  |
|  | n | 347 | 216 |  | 55 |  | 66 |  |  |
| What Happened After the First Visit | The specialist doctor fixed the II appointment, giving the medical prescription (%) | 91.8 | 93.7 |  | 86.0 |  | 90.2 |  |  |
|  | The specialist doctor fixed the II appointment, suggesting that the referring doctor has to prepare the medical prescription (%) | 3.4 | 3.7 |  | 2.3 |  | 2.0 |  |  |
|  | The specialist doctor suggested the II appointment. HCV patient had to book it (%) | 3.1 | 2.1 |  | 7.0 |  | 3.9 |  |  |
|  | The specialist doctor did not say about the II appointment (%) | 1.7 | 0.5 |  | 4.7 |  | 3.9 |  |  |
|  | Overall Analysis |  |  |  |  |  |  |  | χ2(2)=3.374  p=0.185 |
|  | n | 292 | 191 |  | 43 |  | 51 |  |  |

**Supplementary Table 2**. *Referring and specialist doctor: care pathways followed by the HCV patient*. Data refer to whole population, as well as to relative groups arriving to HCV diagnosis through general (A), SERD/prison (B) or specialist (C) physicians. Comparisons between different groups were performed by Kruskal-Wallis analysis. When the overall analysis was statistically significant, a Kruskal-Wallis test was applied again for each category represented.

|  | | Whole Population | A.  General Practitioner | B. SERD/Prison Doctor | C. Opportunistic/Specialistic Doctor | Overall Analysis  (Kruskal-Wallis analysis) |
| --- | --- | --- | --- | --- | --- | --- |
| n | | 347 | 216 | 55 | 66 |  |
| 1. **Referring Doctor - Patient Relationship** | | | | | | |
| Feeling Followed in the Process | 1_Strongly Disagree (%) | 6.9 | 4.9 | 4.9 | 12.3 |  |
|  | 2_Disagree (%) | 3.1 | 2.7 | 7.3 | 1.8 |  |
|  | 3_Undecided (%) | 9.3 | 9.2 | 9.8 | 10.5 |  |
|  | 4_Agree (%) | 14.4 | 15.2 | 12.2 | 14.0 |  |
|  | 5_Strongly Agree (%) | 66.3 | 67.9 | 65.9 | 61.4 |  |
|  | Mean Ranks |  | 144.69 | 139.76 | 132.46 | χ2(2)=1.419  p=0.492 |
|  | Mean±SD | 4.30±1.19 | 4.39±1.09 | 4.27±1.21 | 4.11±1.39 |  |
|  | n | 291 | 184 | 41 | 57 |  |
| Clear Explanation of the Disease | 1_Strongly Disagree (%) | 7.8 | 6.9 | 2.4 | 12.5 |  |
|  | 2_Disagree (%) | 3.4 | 3.2 | 0.0 | 5.4 |  |
|  | 3_Undecided (%) | 7.5 | 8.0 | 7.3 | 7.1 |  |
|  | 4_Agree (%) | 10.6 | 11.2 | 9.8 | 8.9 |  |
|  | 5_Strongly Agree (%) | 70.6 | 70.7 | 80.5 | 66.1 |  |
|  | Mean Ranks |  | 142.61 | 157.99 | 133.33 | χ2(2)=3.348  p=0.188 |
|  | Mean±SD | 4.33±1.23 | 4.36±1.19 | 4.66±0.83 | 4.11±1.45 |  |
|  | n | 293 | 188 | 41 | 56 |  |
| Availability | 1_Strongly Disagree (%) | 4.2 | 2.7 | 2.6 | 8.9 |  |
|  | 2_Disagree (%) | 3.2 | 2.2 | 5.1 | 5.4 |  |
|  | 3_Undecided (%) | 9.5 | 8.8 | 7.7 | 10.7 |  |
|  | 4_Agree (%) | 11.2 | 11.5 | 15.4 | 8.9 |  |
|  | 5_Strongly Agree (%) | 71.9 | 74.7 | 69.2 | 66.1 |  |
|  | Mean Ranks |  | 143.15 | 135.86 | 127.70 | χ2(2)=2.678  p=0.262 |
|  | Mean±SD | 4.44±1.07 | 4.53±0.94 | 4.44±1.02 | 4.18±1.34 |  |
|  | n | 285 | 182 | 39 | 56 |  |
| Help to Face Fears | 1_Strongly Disagree (%) | 7.5 | 5.3 | 11.1 | 11.8 |  |
|  | 2_Disagree (%) | 3.8 | 2.9 | 2.8 | 7.8 |  |
|  | 3_Undecided (%) | 10.2 | 12.9 | 8.3 | 3.9 |  |
|  | 4_Agree (%) | 13.2 | 11.7 | 16.7 | 13.7 |  |
|  | 5_Strongly Agree (%) | 65.4 | 67.3 | 61.1 | 62.7 |  |
|  | Mean Ranks |  | 132.33 | 123.88 | 123.98 | χ2(2)=1.020  p=0.600 |
|  | Mean±SD | 4.25±1.24 | 4.33±1.14 | 4.14±1.36 | 4.08±1.44 |  |
|  | n | 266 | 171 | 36 | 51 |  |
| Feeling Involved in Choices of the Treatment Path | 1_Strongly Disagree (%) | 8.2 | 6.6 | 7.7 | 13.5 |  |
|  | 2_Disagree (%) | 2.9 | 2.8 | 2.6 | 3.8 |  |
|  | 3_Undecided (%) | 8.2 | 9.9 | 2.6 | 5.8 |  |
|  | 4_Agree (%) | 11.1 | 11.6 | 10.3 | 9.6 |  |
|  | 5_Strongly Agree (%) | 69.6 | 69.1 | 76.9 | 67.3 |  |
|  | Mean Ranks |  | 136.04 | 146.09 | 130.92 | χ2(2)=1.290  p=0.525 |
|  | Mean±SD | 4.31±1.24 | 4.34±1.18 | 4.46±1.19 | 4.13±1.46 |  |
|  | n | 280 | 181 | 39 | 52 |  |
| 1. **Specialist Doctor - Patient Relationship** | | | | | | |
| Attentively care in the Treatment Path | 1_Strongly Disagree (%) | 1.0 | 0.6 | 4.5 | 0.0 |  |
|  | 2_Disagree (%) | 0.7 | 0.6 | 2.3 | 0.0 |  |
|  | 3_Undecided (%) | 3.8 | 3.4 | 9.1 | 1.7 |  |
|  | 4_Agree (%) | 11.0 | 9.6 | 9.1 | 16.7 |  |
|  | 5_Strongly Agree (%) | 83.5 | 86.0 | 75.0 | 81.7 |  |
|  | Mean Ranks |  | 145.29 | 127.85 | 140.27 | χ2(2)=3.883  p=0.144 |
|  | Mean±SD | 4.75±4.75 | 4.8±0.58 | 4.48±1.07 | 4.8±0.44 |  |
|  | n | 291 | 178 | 44 | 60 |  |
| Disease: Clear and Complete Information | 1_Strongly Disagree (%) | 0.7 | 1.1 | 0.0 | 0.0 |  |
|  | 2_Disagree (%) | 0.7 | 0.5 | 2.6 | 0.0 |  |
|  | 3_Undecided (%) | 3.5 | 3.8 | 5.1 | 1.7 |  |
|  | 4_Agree (%) | 9.7 | 8.8 | 7.7 | 13.3 |  |
|  | 5_Strongly Agree (%) | 85.5 | 85.7 | 84.6 | 85.0 |  |
|  | Mean Ranks |  | 141.29 | 139.38 | 141.17 | χ2(2)=0.048  p=0.976 |
|  | Mean±SD | 4.79±4.8 | 4.77±0.65 | 4.74±0.68 | 4.83±0.42 |  |
|  | n | 289 | 182 | 39 | 60 |  |
| Therapy: Clear and Complete Information | 1_Strongly Disagree (%) | 0.7 | 1.1 | 0.0 | 0.0 |  |
|  | 2_Disagree (%) | 0.0 | 0.0 | 0.0 | 0.0 |  |
|  | 3_Undecided (%) | 2.1 | 1.7 | 4.9 | 1.7 |  |
|  | 4_Agree (%) | 11.6 | 12.0 | 12.2 | 11.7 |  |
|  | 5_Strongly Agree (%) | 85.6 | 85.1 | 82.9 | 86.7 |  |
|  | Mean Ranks |  | 138.49 | 135.17 | 140.81 | χ2(2)=0.319  p=0.853 |
|  | Mean±SD | 4.81±4.84 | 4.8±0.58 | 4.78±0.52 | 4.85±0.4 |  |
|  | n | 284 | 175 | 41 | 60 |  |
| Therapy Duration: Clear and Complete Information | 1_Strongly Disagree (%) | 1.4 | 2.2 | 0.0 | 0.0 |  |
|  | 2_Disagree (%) | 0.4 | 0.6 | 0.0 | 0.0 |  |
|  | 3_Undecided (%) | 2.5 | 1.7 | 7.9 | 1.7 |  |
|  | 4_Agree (%) | 8.8 | 7.8 | 10.5 | 11.9 |  |
|  | 5_Strongly Agree (%) | 87.0 | 87.8 | 81.6 | 86.4 |  |
|  | Mean Ranks |  | 140.43 | 131.83 | 139.26 | χ2(2)=1.039  p=0.595 |
|  | Mean±SD | 4.8±4.7 | 4.78±0.71 | 4.74±0.6 | 4.85±0.41 |  |
|  | n | 284 | 180 | 38 | 59 |  |
| Adverse Effects: Clear and Complete Information | 1_Strongly Disagree (%) | 1.8 | 2.3 | 0.0 | 1.7 |  |
|  | 2_Disagree (%) | 0.0 | 0.0 | 0.0 | 0.0 |  |
|  | 3_Undecided (%) | 2.9 | 3.4 | 5.4 | 0.0 |  |
|  | 4_Agree (%) | 11.9 | 10.3 | 13.5 | 15.5 |  |
|  | 5_Strongly Agree (%) | 83.4 | 83.9 | 81.1 | 82.8 |  |
|  | Mean Ranks |  | 135.60 | 132.20 | 134.97 | χ2(2)=0.139  p=0.933 |
|  | Mean±SD | 4.75±4.72 | 4.74±0.74 | 4.76±0.55 | 4.78±0.62 |  |
|  | n | 277 | 174 | 37 | 58 |  |
| Attended Result of the Therapy: Clear and Complete Information | 1_Strongly Disagree (%) | 0.7 | 1.2 | 0.0 | 0.0 |  |
|  | 2_Disagree (%) | 0.0 | 0.0 | 0.0 | 0.0 |  |
|  | 3_Undecided (%) | 2.5 | 2.3 | 7.9 | 0.0 |  |
|  | 4_Agree (%) | 12.3 | 12.2 | 10.5 | 13.8 |  |
|  | 5_Strongly Agree (%) | 84.4 | 84.3 | 81.6 | 86.2 |  |
|  | Mean Ranks |  | 134.42 | 130.07 | 137.64 | χ2(2)=0.551  p=0.759 |
|  | Mean±SD | 4.80±0.00 | 4.78±0.6 | 4.74±0.6 | 4.86±0.35 |  |
|  | n | 276 | 172 | 38 | 58 |  |
| Availability for Doubts and Questions | 1_Strongly Disagree (%) | 0.4 | 0.6 | 0.0 | 0.0 |  |
|  | 2_Disagree (%) | 0.0 | 0.0 | 0.0 | 0.0 |  |
|  | 3_Undecided (%) | 2.5 | 2.3 | 7.9 | 0.0 |  |
|  | 4_Agree (%) | 9.7 | 8.7 | 7.9 | 13.6 |  |
|  | 5_Strongly Agree (%) | 87.4 | 88.4 | 84.2 | 86.4 |  |
|  | Mean Ranks |  | 136.86 | 130.51 | 134.74 | χ2(2)=0.642  p=0.725 |
|  | Mean±SD | 4.84±4.75 | 4.84±0.5 | 4.76±0.59 | 4.86±0.35 |  |
|  | n | 278 | 173 | 38 | 59 |  |
| Help with Fears and Anxieties | 1_Strongly Disagree (%) | 2.2 | 1.8 | 5.6 | 1.8 |  |
|  | 2_Disagree (%) | 0.7 | 1.2 | 0.0 | 0.0 |  |
|  | 3_Undecided (%) | 3.3 | 3.6 | 2.8 | 3.5 |  |
|  | 4_Agree (%) | 12.6 | 11.9 | 11.1 | 17.5 |  |
|  | 5_Strongly Agree (%) | 81.0 | 81.5 | 80.6 | 77.2 |  |
|  | Mean Ranks |  | 132.35 | 130.46 | 127.38 | χ2(2)=0.391  p=0.822 |
|  | Mean±SD | 4.7±4.79 | 4.7±0.75 | 4.61±0.99 | 4.68±0.71 |  |
|  | n | 269 | 168 | 36 | 57 |  |
| Involvement in Choices of the Treatment Path | 1_Strongly Disagree (%) | 1.4 | 1.2 | 2.6 | 1.7 |  |
|  | 2_Disagree (%) | 0.7 | 0.6 | 2.6 | 0.0 |  |
|  | 3_Undecided (%) | 3.6 | 4.1 | 5.1 | 1.7 |  |
|  | 4_Agree (%) | 12.3 | 10.5 | 12.8 | 17.2 |  |
|  | 5_Strongly Agree (%) | 81.9 | 83.6 | 76.9 | 79.3 |  |
|  | Mean Ranks |  | 136.97 | 127.33 | 132.03 | χ2(2)=1.251  p=0.535 |
|  | Mean±SD | 4.72±4.81 | 4.75±0.67 | 4.59±0.91 | 4.72±0.67 |  |
|  | n | 276 | 171 | 39 | 58 |  |

**Supplementary Table 3.** *PREMs: Patients' feedback on referring (I) and specialist doctors (II) at T0.* Data refer to whole population, as well as to relative groups arriving to HCV diagnosis through general (A), SERD/prison (B) or specialist (C) physicians. Comparisons between different groups were performed by Kruskal-Wallis analysis.

|  | | Whole Population | A.  General Practitioner | A  vs  B | B. SERD/Prison Doctor | B  vs  C | C. Opportunistic/Specialistic Doctor | C  vs  A | Overall Analysis  (Kruskal-Wallis analysis) |
| --- | --- | --- | --- | --- | --- | --- | --- | --- | --- |
|  | n | 347 | 216 |  | 55 |  | 66 |  |  |
| Health Status | 1_Poor (%) | 9.0 | 8.7 |  | 11.5 |  | 7.6 |  |  |
|  | 2_Passable (%) | 28.1 | 24.0 |  | 34.6 |  | 33.3 |  |  |
|  | 3_Good (%) | 46.9 | 51.0 |  | 38.5 |  | 43.9 |  |  |
|  | 4_Very good (%) | 12.8 | 12.0 |  | 11.5 |  | 15.2 |  |  |
|  | 5_Excellent (%) | 3.3 | 4.3 |  | 3.8 |  | 0.0 |  |  |
|  | Mean Ranks |  | 168.95 |  | 149.67 |  | 157.23 |  | χ2(2)=2.424  p=0.298 |
|  | Mean±SD | 2.73±0.91 | 2.79±0.92 |  | 2.62±0.97 |  | 2.67±0.83 |  |  |
|  | n | 335 | 208 |  | 52 |  | 66 |  |  |
| Limitations to Moderate Physical Activities | 1_Yes, quite a lot (%) | 15.2 | 13.3 |  | 15.4 |  | 18.5 |  |  |
|  | 2_Yes, partially (%) | 25.2 | 22.7 |  | 32.7 |  | 26.2 |  |  |
|  | 3_No (%) | 59.6 | 64.0 |  | 51.9 |  | 55.4 |  |  |
|  | Mean Ranks |  | 166.47 |  | 148.61 |  | 151.38 |  | χ2(2)=3.066  p=0.216 |
|  | Mean±SD | 2.44±0.74 | 2.51±0.72 |  | 2.37±0.74 |  | 2.37±0.78 |  |  |
|  | n | 329 | 203 |  | 52 |  | 65 |  |  |
| Limitations in Climbing Stairs | 1_Yes, quite a lot (%) | 14.2 | 12.1 |  | 11.5 |  | 20.0 |  |  |
|  | 2_Yes, partially (%) | 27.1 | 28.0 |  | 30.8 |  | 24.6 |  |  |
|  | 3_No (%) | 58.7 | 59.9 |  | 57.7 |  | 55.4 |  |  |
|  | Mean Ranks |  | 165.27 |  | 162.54 |  | 153.64 |  | χ2(2)=0.985  p=0.611 |
|  | Mean±SD | 2.45±0.73 | 2.48±0.70 |  | 2.46±0.70 |  | 2.35±0.80 |  |  |
|  | n | 332 | 207 |  | 52 |  | 65 |  |  |
| Last 4 Weeks: Work/Daily Lower Performance due Heath Status | Yes (%) | 31. 5 | 26.6 |  | 41.2 |  | 38.5 |  | χ2(2)=5.845  p=0.054 |
|  | No (%) | 68.5 | 73.4 |  | 58.8 |  | 61.5 |  |  |
|  | n | 324 | 199 |  | 51 |  | 65 |  |  |
| Last 4 Weeks: Work or Daily Limitations | Yes (%) | 30.2 | 24.3 | 0.254 | 36.5 | 1.000 | 41.5 | **0.024** | **χ2(2)=8.353**  **p=0.015** |
|  | No (%) | 69.8 | 75.7 |  | 63.5 |  | 58.5 |  |  |
|  | n | 328 | 202 |  | 52 |  | 65 |  |  |
| Last 4 Weeks: Work/Daily Lower Performance due to Emotional State | Yes (%) | 32.8 | 27.3 | **0.014** | 48.1 | 0.818 | 38.5 | 0.290 | **χ2(2)=9.129**  **p=0.010** |
|  | No (%) | 67.2 | 72.7 |  | 51.9 |  | 61.5 |  |  |
|  | n | 323 | 198 |  | 52 |  | 65 |  |  |
| Last 4 Weeks: Work/Daily Decreased Concentration due to Emotional State | Yes (%) | 29.5 | 24.5 | **0.028** | 43.1 | 0.830 | 33.8 | 0.455 | **χ2(2)=7.541**  **p=0.023** |
|  | No (%) | 70.5 | 75.5 |  | 56.9 |  | 66.2 |  |  |
|  | n | 325 | 200 |  | 51 |  | 65 |  |  |
| Last 4 Weeks: Work/Daily Activities Limitations Due to Pain | 1_Very much (%) | 2.1 | 1.0 |  | 3.8 |  | 1.5 |  |  |
|  | 2_Very (%) | 7.6 | 5.9 |  | 7.7 |  | 12.3 |  |  |
|  | 3_A bit (%) | 18.8 | 18.6 |  | 21.2 |  | 20.0 |  |  |
|  | 4_Very little (%) | 18.5 | 19.6 |  | 17.3 |  | 16.9 |  |  |
|  | 5_For nothing (%) | 53.0 | 54.9 |  | 50.0 |  | 49.2 |  |  |
|  | Mean Ranks |  | 166.00 |  | 153.54 |  | 151.29 |  | χ2(2)=1.956  p=0.376 |
|  | Mean±SD | 4.13±1.10 | 4.22±1.01 |  | 4.02±1.18 |  | 4.00±1.16 |  |  |
|  | n | 330 | 204.0 |  | 52.0 |  | 65.0 |  |  |
| Last 4 Weeks: How Often Calm/Serene | 1_Never (%) | 1.2 | 1.0 |  | 2.0 |  | 1.6 |  |  |
|  | 2_Almost never (%) | 11.9 | 8.7 |  | 20.0 |  | 17.7 |  |  |
|  | 3_Part of the time (%) | 29.7 | 28.0 |  | 34.0 |  | 32.3 |  |  |
|  | 4_A long time (%) | 9.8 | 8.7 |  | 12.0 |  | 12.9 |  |  |
|  | 5_Almost always (%) | 33.0 | 36.7 |  | 18.0 |  | 29.0 |  |  |
|  | 6_Always (%) | 14.4 | 16.9 |  | 14.0 |  | 6.5 |  |  |
|  | Mean Ranks |  | 172.68 | **0.026** | 135.80 | 1.000 | 137.17 | **0.018** | **χ2(2)=11.943**  **p=0.003** |
|  | Mean±SD | 4.05±1.34 | 4.22±1.31 |  | 3.66±1.41 |  | 3.69±1.29 |  |  |
|  | n | 327 | 207 |  | 50 |  | 62.0 |  |  |
| Last 4 Weeks: How Often Full of Energy | 1_Never (%) | 3.7 | 2.9 |  | 9.8 |  | 1.6 |  |  |
|  | 2_Almost never (%) | 18.0 | 14.1 |  | 19.6 |  | 28.6 |  |  |
|  | 3_Part of the time (%) | 31.8 | 31.7 |  | 33.3 |  | 33.3 |  |  |
|  | 4_A long time (%) | 8.9 | 9.8 |  | 7.8 |  | 7.9 |  |  |
|  | 5_Almost always (%) | 28.7 | 32.2 |  | 21.6 |  | 23.8 |  |  |
|  | 6_Always (%) | 8.9 | 9.3 |  | 7.8 |  | 4.8 |  |  |
|  | Mean Ranks |  | 170.69 | 0.102 | 141.10 | 1.000 | 140.52 | 0.057 | **χ2(2)=8.241**  **p=0.016** |
|  | Mean±SD | 3.68±1.38 | 3.82±1.35 |  | 3.35±1.47 |  | 3.38±1.30 |  |  |
|  | n | 327 | 205 |  | 51 |  | 63 |  |  |
| Last 4 Weeks: How Long Discouraged/Sad | 1_Never (%) | 2.8 | 2.9 |  | 4.0 |  | 1.6 |  |  |
|  | 2_Almost never (%) | 9.5 | 6.8 |  | 12.0 |  | 15.9 |  |  |
|  | 3_Part of the time (%) | 5.8 | 4.9 |  | 8.0 |  | 7.9 |  |  |
|  | 4_A long time (%) | 33.6 | 31.6 |  | 38.0 |  | 38.1 |  |  |
|  | 5_Almost always (%) | 29.7 | 34.0 |  | 20.0 |  | 25.4 |  |  |
|  | 6_Always (%) | 18.7 | 19.9 |  | 18.0 |  | 11.1 |  |  |
|  | Mean Ranks |  | 170.49 | 0.209 | 145.09 | 1.000 | 137.52 | **0.030** | **χ2(2)=8.320**  **p=0.016** |
|  | Mean±SD | 4.34±1.27 | 4.47±1.23 |  | 4.12±1.36 |  | 4.03±1.26 |  |  |
|  | n | 327 | 206 |  | 50 |  | 63 |  |  |
| Last 4 Weeks: Limits on Social Activities | 1_Always (%) | 2.8 | 2.0 |  | 6.0 |  | 3.2 |  |  |
|  | 2_Almost always (%) | 13.8 | 9.8 |  | 26.0 |  | 17.5 |  |  |
|  | 3_Part of the time (%) | 26.4 | 23.4 |  | 34.0 |  | 31.7 |  |  |
|  | 4_Almost never (%) | 27.9 | 32.2 |  | 12.0 |  | 25.4 |  |  |
|  | 5_Never (%) | 29.1 | 32.7 |  | 22.0 |  | 22.2 |  |  |
|  | Mean Ranks |  | 173.47 | **0.001** | 122.98 | 0.699 | 143.04 | 0.052 | **χ2(2)=15.692**  **p<0.0005** |
|  | Mean±SD | 3.67±1.12 | 3.84±1.05 |  | 3.18±1.22 |  | 3.46±1.12 |  |  |
|  | n | 326 | 205 |  | 50 |  | 63 |  |  |

**Supplementary Table 4.** *PROMs: Patients' feedback on outcome at T0.* Data refer to whole population, as well as to relative groups arriving to HCV diagnosis through general (A), SERD/prison (B) or specialist (C) physicians. Comparisons between different groups were performed by Kruskal-Wallis analysis.

|  |  | Whole Population | A.  General Practitioner | A  vs  B | B. SERD/Prison Doctor | B  vs  C | C. Opportunistic/ Specialistic Doctor | C  vs  A | Overall Analysis  (Kruskal-Wallis analysis) |
| --- | --- | --- | --- | --- | --- | --- | --- | --- | --- |
|  | n | 347 | 216 |  | 55 |  | 66 |  |  |
| Support Along the Path | 1_Strongly Disagree (%) | 0.9 | 0.7 |  | 3.1 |  | 0.0 |  |  |
|  | 2_Disagree (%) | 0.4 | 0.7 |  | 0.0 |  | 0.0 |  |  |
|  | 3_Undecided (%) | 2.6 | 2.7 |  | 3.1 |  | 2.0 |  |  |
|  | 4_Agree (%) | 18.7 | 15.5 |  | 12.5 |  | 32.7 |  |  |
|  | 5_Strongly Agree (%) | 77.4 | 80.4 |  | 81.3 |  | 65.3 |  |  |
|  | Mean Ranks |  | 118.40 |  | 118.69 |  | 102.34 |  | χ2(2)=4.285  p=0.117 |
|  | Mean±SD | 4.71±0.62 | 4.74±0.61 |  | 4.69±0.82 |  | 4.63±0.53 |  |  |
|  | n | 235 | 148 |  | 32 |  | 49 |  |  |
| Know who to contact in case of need | 1_Strongly Disagree (%) | 2.2 | 1.4 |  | 3.2 |  | 2.1 |  |  |
|  | 2_Disagree (%) | 0.9 | 1.4 |  | 0.0 |  | 0.0 |  |  |
|  | 3_Undecided (%) | 3.1 | 2.8 |  | 3.2 |  | 4.2 |  |  |
|  | 4_Agree (%) | 15.9 | 14.1 |  | 12.9 |  | 22.9 |  |  |
|  | 5_Strongly Agree (%) | 78.0 | 80.3 |  | 80.6 |  | 70.8 |  |  |
|  | Mean Ranks |  | 113.14 |  | 113.27 |  | 103.20 |  | χ2(2)=1.768  p=0.413 |
|  | Mean±SD | 4.67±0.77 | 4.7±0.72 |  | 4.68±0.83 |  | 4.6±0.76 |  |  |
|  | n | 227 | 142 |  | 31 |  | 48 |  |  |
| Recommend Primary Care Services | 1_Strongly Disagree (%) | 2.2 | 1.4 |  | 3.2 |  | 4.2 |  |  |
|  | 2_Disagree (%) | 0.9 | 0.7 |  | 0.0 |  | 2.1 |  |  |
|  | 3_Undecided (%) | 3.6 | 4.3 |  | 3.2 |  | 2.1 |  |  |
|  | 4_Agree (%) | 12.9 | 7.9 |  | 16.1 |  | 25.0 |  |  |
|  | 5_Strongly Agree (%) | 80.4 | 85.6 |  | 77.4 |  | 66.7 |  |  |
|  | Mean Ranks |  | 115.07 | 0.996 | 106.65 | 0.769 | 95.21 | **0.020** | **χ2(2)=7.518**  **p=0.023** |
|  | Mean±SD | 4.68±0.78 | 4.76±0.70 |  | 4.65±0.84 |  | 4.48±0.97 |  |  |
|  | n | 224 | 139 |  | 31 |  | 48 |  |  |
| Recommend Specialist Medical Services | 1_Strongly Disagree (%) | 0.5 | 0.0 |  | 0.0 |  | 2.1 |  |  |
|  | 2_Disagree (%) | 0.5 | 0.7 |  | 0.0 |  | 0.0 |  |  |
|  | 3_Undecided (%) | 1.9 | 2.2 |  | 3.4 |  | 0.0 |  |  |
|  | 4_Agree (%) | 10.6 | 6.7 |  | 10.3 |  | 20.8 |  |  |
|  | 5_Strongly Agree (%) | 86.6 | 90.3 |  | 86.2 |  | 77.1 |  |  |
|  | Mean Ranks |  | 109.65 |  | 105.41 |  | 96.17 |  | χ2(2)=4.983  p=0.083 |
|  | Mean±SD | 4.82±0.52 | 4.87±0.46 |  | 4.83±0.47 |  | 4.71±0.68 |  |  |
|  | n | 216 | 134 |  | 29 |  | 48 |  |  |

**Supplementary Table 5.** *PREMs: overall experience along the care pathway at T1*. Data refer to whole population, as well as to relative groups arriving to HCV diagnosis through general (A), SERD/prison (B) or specialist (C) physicians. Comparisons between different groups were performed by Kruskal-Wallis analysis.
