# Supplementary material for: Clinical outcomes and patient-reported measures in HCV care: Insight from a longitudinal prospective study in a large Italian region
Source: PLoS One. 2026 Mar 11;21(3):e0343936. doi: 10.1371/journal.pone.0343936 (PMC12978477; doi:10.1371/journal.pone.0343936)
Supplement: Attachment — Submitted filename: HCV rev.docx [file pone.0343936.s002.docx]

Dear sir,

thank you to select me to review a manuscript: Clinical outcomes and patient-reported measures in HCV care: insight from a longitudinal prospective study in a large Italian region; written by Moretti et al. Authors evaluated clinical and patient-reported outcomes in the HCV care cascade in Tuscany (Italy), offering insights into how health service organization affects effectiveness, equity, and patient experience. 963 patients with chonic hepatitis C from 7 hepatology or infectology centers were treated with DAA; the mean age was 56.31±15.57 years, 61.9% were males. 17.5% had a history of detention, 2.6% were current detainees. 93.4% of patients achieved SVR. The authors evaluated patient-reported outcome measures (PROMs) and patient-reported experience measures (PREMs). PROMs and PREMs were collected at baseline (T0), 3 months post-treatment (T1), and 6 months after T1 (T2). The number of patients that filled the questionnaires were 347, 237, and 104 at T0, T1 and T2, respectively. Patients which filled the T0 questionnaire were older and had less frequent loss of follow-up.

Relative to referring doctor, a significant improvement was observed in all PREMs from T0 to T1. These improvements were generally maintained at T2, except for feelings of being attentively cared by medical doctors, which showed a slight decrease. No significant temporal differences were observed in the PREMs relative to the specialist doctor. HCV patients showed a significant improvement in perceived health status from T0 to T1 (p=0.001), which remained stable over time. Significant differences were observed between T0 and T2: limitations in work/daily concentration and performance due to emotional state or health status.

The specialist doctor group had a significantly higher proportion of patients who were directly referred to specialized HCV treatment facilities at the time of diagnosis (80%), compared to the GP group (61.6%, p=0.008).

SERD/prison services were younger and mostly male. They had higher psychiatric comorbidities and risk behaviors. Patients identified by SERD/prison doctors were notably younger (mean age: 45.89 ± 11.19 years), with higher prevalence of males (90.9%), unemployed (47.1%) and more likely to engage in high-risk behaviors, such as unprotected sex (96.1%) and injection drug use (90.6%). Psychiatric disorders were significantly more prevalent, affecting 33.3% of patients.

Manuscript is well written, abstract and introduction are adequate. Statistical analysis is well made, results are clearly presented. Major limitation of this interesting study is a small number of patients who completed questionairres. Discussion is too long and not always targeted to the topic of the research.

I recommend some changes to improve the quality of this nice manuscript:

1) Please, explain in detail a small number of patients in T0, T1 and T2 group. Please explain the very low number of patients in group T2 and the big decrease in respondents between groups T1 and T2.
2) Please specify which DAA regimen patients were treated with.
3) Please replace the term echography with abdominal ultrasound.
4) Please specify, how the fibrosis stage was diagnosed.
5) Please shorten the discussion.
6) English language pollishing is recommended.

My final decision is minor revision.
